# Exome sequencing (ES) of a pediatric cohort with chronic endocrine diseases: a single-center study (within the framework of the TRANSLATE-NAMSE project)

**DOI:** 10.1007/s12020-023-03581-7

**Published:** 2023-11-08

**Authors:** Sebastian Gippert, Matias Wagner, Theresa Brunet, Riccardo Berruti, Melanie Brugger, Eva M. C. Schwaibold, Tobias B. Haack, Georg F. Hoffmann, Markus Bettendorf, Daniela Choukair

**Affiliations:** 1 Division of Pediatric Endocrinology and Diabetes, Center for Pediatrics and Adolescent Medicine, University Hospital Heidelberg, Heidelberg, Germany and Center for Rare Diseases, University Hospital Heidelberg, Heidelberg, Germany; 2 Institute of Human Genetics, Klinikum rechts der Isar, School of Medicine, Technical University of Munich, Munich, Germany; 3 Institute for Neurogenomics, Helmholtz Zentrum München, Neuherberg, Germany; 4 Department of Pediatric Neurology and Developmental Medicine, Hauner Children’s Hospital, Ludwig Maximilian University of Munich, Munich, Germany; 5 Institute of Human Genetics, Heidelberg University, Heidelberg, Germany; 6 Institute of Medical Genetics and Applied Genomics, University of Tuebingen, Tübingen, Germany and Centre for Rare Diseases, University of Tuebingen, Tübingen, Germany

**Keywords:** Exome sequencing, chronic pediatric endocrine diseases, TRANSLATE-NAMSE, rare diseases, multidisciplinary case conferences

## Abstract

**Background:**

Endocrine disorders are heterogeneous and include a significant number of rare monogenic diseases.

**Methods:**

We performed exome sequencing (ES) in 106 children recruited from a single center within the TRANSLATE‑NAMSE project. They were categorized into subgroups: proportionate short stature (PSS), disproportionate short stature (DSS), hypopituitarism (H), differences in sexual development (DSD), syndromic diseases (SD) and others.

**Results:**

The overall diagnostic yield was 34.9% (*n* = 37/106), including 5 patients with variants in candidate genes, which have contributed to collaborations to identify gene-disease associations. The diagnostic yield varied significantly between subgroups: PSS: 16.6% (1/6); DSS: 18.8% (3/16); H: 17.1% (6/35); DSD: 37.5% (3/8); SD: 66.6% (22/33); others: 25% (2/8). Confirmed diagnoses included 75% ultrarare diseases. Three patients harbored more than one disease-causing variant, resulting in dual diagnoses.

**Conclusions:**

ES is an effective tool for genetic diagnosis in pediatric patients with complex endocrine diseases. An accurate phenotypic description, including comprehensive endocrine diagnostics, as well as the evaluation of variants in multidisciplinary case conferences involving geneticists, are necessary for personalized diagnostic care. Here, we illustrate the broad spectrum of genetic endocrinopathies that have led to the initiation of specific treatment, surveillance, and family counseling.

**Graphical Abstract:**

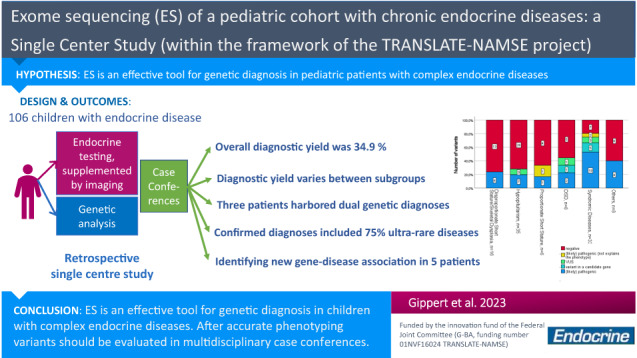

## Background

Endocrine disorders comprise a wide spectrum of different diseases (affecting the hypothalamus and pituitary, thyroid gland, adrenal cortex, sexual differentiation and growth disorders, polyendocrine and neoplastic disorders) [1]. Autoimmune disorders, environmental factors or medications can lead to endocrine dysfunction, but genetic etiology contributes significantly to the spectrum of pediatric endocrine disorders.

In recent years, the use of next-generation sequencing, such as exome sequencing (ES), has increased significantly in almost every medical specialty. However, to the best of our knowledge, there are few data on genetic diagnoses by ES in a representative endocrine cohort from a single center.

To improve the care of patients with rare diseases, the German Federal Joint Committee (G-BA) funded the innovation project TRANSLATE-NAMSE from April 2017 to September 2020 [2].

TRANSLATE-NAMSE was a healthcare project to establish new structures and processes across different healthcare providers and disciplines. Ten German centers for rare diseases (Berlin, Bonn, Bochum, Dresden, Essen, Hamburg, Heidelberg, Luebeck, Munich, Tuebingen), two health insurance companies (AOK Nordost; Barmer GEK) and the Alliance for Chronic Rare Diseases (ACHSE e.V.) formed a consortium to design, test and evaluate a model of structured care for patients with rare diseases [2]. Here, in a retrospective single-center study, we elucidated the spectrum of genetic variations underlying rare endocrine diseases. We describe in detail the clinical and genetic findings of 106 children and adolescents with endocrine disorders who underwent ES.

## Materials and Methods

### Study design

Children and adolescents with various endocrine diseases were recruited consecutively from December 2017 to February 2020 within the framework of the healthcare project TRANSLATE-NAMSE in the endocrinological outpatient clinic of the University Children´s Hospital in Heidelberg [2]. The analysis of the results of ES was conducted under the guidelines of the Ethics Committee of the University of Heidelberg (S-690/2020) and in accordance with the current version of the Declaration of Helsinki (2013). Initially, baseline and dynamic endocrine testing were performed, possibly supplemented by imaging procedures. Inclusion criteria were a) an endocrine disorder or symptom complex with at least one endocrine disorder, b) no established genetic diagnosis and c) no alternative causal explanation for the condition, such as an autoimmune disease. These patients were divided into subgroups: proportionate short stature (PSS), disproportionate short stature (DSS), hypopituitarism (H), differences in sexual development (DSD), syndromic diseases (SD), and others. For detailed clinical characteristics, see Table 1. Most exome analyses were performed as trio-sequencing (parent‒child-trios; *n* = 72, 67.9%) followed by single-analyses (*n* = 26, 24.5%). Duo-exomes were performed in 5 patients (4.7%), and quattro-exomes were performed in 3 patients (2.8%). Phenotypes were compiled using Human Phenotype Ontology (HPO) terms [3].

**Table 1:** Clinical characterization (*n* = 106)

	Proportionate Short Stature
N (male)	6 (4)
Mean age ± SD (years)	11.3 ± 5.6
	**Disproportionate Short Stature**
N (male)	16 (9)
Mean age ± SD (years)	10.5 ± 3.7
	**Hypopituitarism**
N (male)	35 (24)
Mean age ± SD (years)	10.2 ± 6.5
	*Hypophyseal hormone deficiencies (n)*
GH	15
LH/FSH	5
ACTH	3 (1^a^)
AVP	9
TSH + PRL	1
ACTH + GH	2 (1^a^)
ACTH + GH + TSH	3
GH + TSH + LH/FSH	1
ACTH + GH + TSH + LH/FSH	4
	**Differences of sexual development (DSD)**
N (male)	8 (6)
Mean age ± SD (years)	4.3 ± 6.5
	*Karyotypes (n)*
Phenotypic male patients with XY karyotype	6
Phenotypic female patients with male karyotype XY	2
	**Syndromic diseases** ***(SD)***
N (male)	33 (21)
Mean age ± SD (years)	10.2 ± 7.1
	*Endocrine dysfunction (n)*
Short Stature	23
Obesity	3
Tall Stature	3
Hypopituitarism	2
Genital hypoplasia or hypospadias	2
	**Others**
N (male)	8 (4)
Mean age ± SD (years)	6.6 ± 7.1
	*Diagnoses (n)*
Congenital primary hypothyroidism	2
Pseudohypoparathyroidism	1
Septo-optic dysplasia	3
Juvenile osteoporosis	1
Neurofibromatosis	1
Hypophosphatemic rickets	1

^a^Temporary ACTH deficiency.

ES was performed in the laboratories of the Institute of Human Genetics, Technical University Munich, the Institute of Human Genetics, Tuebingen and the Institute of Human Genetics, Heidelberg. All patients received information and care at Heidelberg University Children’s Hospital. The indication and subsequent evaluation of this specific diagnostic tool was made in multidisciplinary case conferences, always including pediatric endocrinologists and geneticists. All patients or their legal guardians gave written consent for both the genetic diagnostics and the TRANSLATE-NAMSE project with retrospective analysis and publication of the data. Unsolved cases with variants in candidate genes were reanalyzed in multidisciplinary case conferences after 2 years.

### Exome sequencing

ES was performed in Munich using the SureSelect Human All Exon Kit (Agilent, 60 Mb V6) for enrichment and a NovaSeq6000 (Illumina, San Diego, CA, USA) for paired-end sequencing. Reads were aligned to the Human Genome Assembly GRCh37 (hg19). Allele frequency estimation was performed using in-house databases and the Genome Aggregation Database (gnomAD). Variants were analyzed with a MAF < 1% (autosomal-recessive inheritance) and >0.01% for de novo variants. In addition, a phenotype-based search was conducted by performing an OMIM full term search using the three most characteristic phenotypic traits to establish a gene list. The filter queries variants with a MAF < 0.1%. Moreover, CNVs with a MAF < 0.01 and mtDNA variants with a MAF < 1% were assessed. Identified variants were classified according to the American College of Medical Genetics and Genomics (ACMG) guidelines [4–6]. In Tuebingen, diagnostic ES and data analysis were performed according to a quality-controlled standard operating procedure essentially as described previously [7]. In brief, coding genomic regions were enriched using a SureSelect XT Human All Exon Kit V.7 (Agilent Technologies, Santa Clara, California, USA) for subsequent sequencing as 2×100 bp paired-end reads on a NovaSeq6000 system (Illumina, San Diego, California, USA). Generated sequences were analyzed using the megSAP pipeline (https://github.com/imgag/megSAP), and prioritized genomic variation (SNPs, indels, CNVs, and SVs) was classified with reference to the ACMG guidelines. One ES was performed at the German Cancer Research Center (DKFZ), Heidelberg, Germany, on DNA of the affected girl and both parents as previously described [8]. All results were evaluated in a multidisciplinary case conference to match the clinical and genetic findings. Only patients with likely pathogenic or pathogenic variants according to ACMG (hereafter referred to as “disease-causing”) in established disease genes were included as solved cases in the overall diagnostic yield. Five individuals with variants in candidate genes subsequently established as disease genes were also categorized as solved and assigned to the overall diagnostic yield. Individuals with (1) negative results (i.e., no variant[s] prioritized), (2) variants of uncertain significance (VUS) in endocrinopathy-associated genes, or (3) variants that did not explain the phenotype were summarized as unsolved patients. The nomenclature of DNA sequence variants was controlled using VariantValidator[9].

### Statistics

Statistical analyses were performed using SPSS Statistics 28.0.1.0 (IBM, Armonk, New York). The results are presented as the mean with standard deviation and median with range. Comparison of the diagnostic yield was performed using Fisher’s exact test and Pearson’s chi-squared test.

## Results

### Patient characteristics

We included a total of 106 patients with a mean age of 9.6 ± 6.5 years (range: 0.1–28.1 years, median: 9.9) at the time of initiation of exome diagnostics during 2017–2020. Sixty-eight were male (64.2%), and 38 were female (35.8%). For clinical characterization, see Table 1 and Supplementary Tables 1 and 2.

### Genetic findings

Exome sequencing initially identified disease-causing variants in 32/106 individuals, representing a diagnostic yield of 30.2%. Five patients had six variants in genes that had not been associated with monogenic disorders at the time of the initial analysis. However, based on international collaborations and subsequent re-evaluation in multidisciplinary conferences, a probable causal association has been established (Table 2). Reanalysis of unsolved patients at 2 years revealed 40 monogenic diagnoses in 37/106 patients due to 3 patients with dual diagnoses. The overall diagnostic yield was 34.9%. Thirty/40 diagnoses (75.0%) were ultrarare diseases with a prevalence < 1 in 50.000 [10].

**Table 2:** List of patients in which the variants could be assigned to be disease-associated (*n* = 37)

Patient	Gene/locus	Variant	Transcript	Inheritance	Variant type	Zygosity	ACMG Variant classification	Diagnosis	OMIM phenotype
	*Disproportionate Short Stature/Skeletal Dysplasia*								
1	*ANKRD11*	c.7534 C > T, p.(Arg2512Trp)	NM_013275.5	Autosomal dominant, de novo	Missense	Heterozygous	Pathogenic	KBG syndrome	#148050
2	*PAPSS2*	c.809 G > A, p.(Gly270Asp)	NM_001015880.1	Autosomal recessive	Missense	Homozygous	Pathogenic	Brachyolmia 4 with mild epiphyseal and metaphyseal changes	#612847
3	*MBTPS1*	c.1995C>G, p.(Tyr665^a^), c.955 G > T, p.(Val319Phe)	NM_003791.2	Autosomal recessive	Nonsense/Missense	Compound-heterozygous	Pathogenic/ likely pathogenic	Spondyloepiphyseal dysplasia, Kondo-Fu type	#618392
	*Hypopituitarism*								
4	*GNRHR*	c.317 A > G, p.(Gln106Arg); c.350 T > G, p.(Leu117Arg)	NM_000406.2	Autosomal recessive	Missense	Compound heterozygous	Pathogenic/ pathogenic	Hypogonadotropic hypogonadism 7 with or without anosmia	#146110
5	*FGFR1*	c.1704+1 G > A, p.(?)	NM_023106.2	Autosomal dominant, de novo	Splice-site	Heterozygous	Pathogenic	Hypogonadotropic hypogonadism 2 with or without anosmia/Kallmann syndrome	#147950
6	*NFKB2*	c.2600 C > T, p.(Ala867Val)	NM_001077494.2	Autosomal dominant, de novo	Missense	Heterozygous	Pathogenic	Immunodeficiency, common variable, 10 (including ACTH deficiency)	#615577
7	*IGSF1*	c.2422dup, p.(His808Profs*14)	NM_001170961.1	X-linked, inherited	Frame-shift	Hemizygous	Pathogenic	Central hypothyroidism with testicular enlargement	#300888
8 und 9	*FGFR1* *Proportionate Short Stature*	c.287 C > G, p.(Ser96Cys)	NM_023110.2	Autosomal dominant, inherited	Missense	Heterozygous	Likely pathogenic	Hypogonadotropic hypogonadism 2 with or without anosmia/Kallmann syndrome 2	#147950
10	*GHR*	c.344 A > C, p.(Asn115Thr)	NM_000163.4	Autosomal recessive	Missense	Homozygous	Likely pathogenic	Laron syndrome	#262500
	*Differences of Sex Development*								
11	*HSD3B2*	c.500 C > T, p.(Ala167Val), c.946 C > T, p.(Arg316Cys)	NM_000198.3	Autosomal recessive	Missense	Homozygous complex allele	Benign/ likely pathogenic	Adrenal hyperplasia, congenital, due to 3-beta-hydroxysteroid dehydrogenase 2 deficiency	#201810
12	*PPP1R12A*^a^	c.2698 C > T, p.(Arg900^a^)	NM_002480.2	Autosomal dominant, de novo	Nonsense	Heterozygous	Pathogenic	Genitourinary and/or brain malformation syndrome	#618820^b^
13	*AR*	c.2495 G > A, p.(Arg832Gln)	NM_000044.5	X-linked, inherited	Missense	Hemizygous	Pathogenic	Androgen Insensitivity syndrome	#300068
	*Syndromic Diseases*								
14	*ABL1*	c.1066 G > A, p.(Ala356Thr)	NM_007313.2	Autosomal dominant, de novo	Missense	Heterozygous	Pathogenic	Congenital heart defects and skeletal malformations syndrome	#617602
15	*ALMS1*	c.4150dup, p.(Gln1384Profs*17)	NM_015120.4	Autosomal recessive	Frame-shift	Homozygous	Pathogenic	Alstrom syndrome	#203800
16	*RAD21*	c.3 G > A, p.(?)	NM_006265.2	Autosomal dominant, de novo	Nonsense	Heterozygous	Pathogenic	Cornelia de Lange syndrome 4 with or without midline brain defects	#614701
17	*FKBP14*	c.636 G > C, p.(*212Tyrext*52)	NM_017946.3	Autosomal recessive	Stop-loss	Homozygous	Pathogenic	Ehlers-Danlos syndrome, kyphoscoliotic type, 2	#614557
18	*PSMD12*	c.148_149del, p.(Leu50Glyfs*26)	NM_002816.3	Autosomal dominant, de novo	Frame-shift	Heterozygous	Pathogenic	Stankiewicz-Isidor syndrome	#617516
19	*PIBF1*	c.1133 A > C, p.(His378Pro), c.1801C>T, p.(Arg601^a^)	NM_006346.2	Autosomal recessive	Missense/Nonsense	Compound heterozygous	Likely pathogenic/ Pathogenic	Joubert syndrome 33	#617767
20	*SMARCA5*^a^	c.1301_1306del, p.(Ile434_Leu435del)	NM 003601.3	Autosomal dominant, de novo	Indel	Heterozygous	Likely pathogenic	Novel	not OMIM listed
21	*POLD1*	c.1812_1814del, p.(Ser605del)	NM_002691.3	Autosomal dominant, de novo	Indel	Heterozygous	Pathogenic	Mandibular hypoplasia, deafness, progeroid features and lipodystrophy syndrome	#615381
22	*BRAF*	c.1741A>G, p.(Asn581Asp)	NM_004333.6	Autosomal dominant, de novo	Missense	Heterozygous	Pathogenic	Noonan syndrome	#613706
23	*PTPN11*	c.922 A > G, p.(Asn308Asp)	NM_002834.5	Autosomal dominant, de novo	Missense	Heterozygous	Pathogenic	Noonan syndrome 1	#163950
24	*PTPN11*	c.794 G > A, p.(Arg265Gln)	NM_002834.5	Autosomal dominant, inherited	Missense	Heterozygous	Pathogenic	Noonan syndrome 1	#163950
25	*DNAJC21*	c.647_666del, p. (Arg216Thrfs*63)	NM_194283.3	Autosomal recessive	Frame-shift	Homozygous	Pathogenic	Bone marrow failure syndrome 3 (Shwachman-Diamond syndrome)	#617052
26	*PUF60*	c.1172_1173insATA, p.(Val392^a^)	NM_078480.2	Autosomal dominant, inherited	Insertion	Heterozygous	Pathogenic	Verheij syndrome	#615583
27 and 28	*ADAMTS10*	c.709 C > T, p.(Arg237^a^)	NM_030957.3	Autosomal recessive	Nonsense	Homozygous	Pathogenic	Weill-Marchesani syndrome	#277600
29	*WRN* *ASPM*	c.3913 C > T, p. p.(Arg1305^a^) c.2474 G > A, p.(Arg825Gln)	NM_000553.4 NM_018136.4	Autosomal recessive Autosomal recessive	Nonsense Missense	Homozygous Homozygous	Pathogenic VUS	Werner syndrome Primary autosomal recessive microcephaly 5	#277700 #608716
30	*KMT2A*	c.1844del, p.(Pro615Argfs*8)	NM_001197104.1	Autosomal dominant, de novo	Frame-shift	Heterozygous	Pathogenic	Wiedemann-Steiner syndrome	#605130
31	*KMT2D*	c.10624 C > G, p.(Leu3542Val)	NM_003482.3	Autosomal dominant, de novo	Missense	Heterozygous	Pathogenic	KMT2D-associated malformation syndrome	#620186
32	*MCM7*^a^	c.776 G > C, p.(Gly259Ala); c.133 C > T, p.(Gln45^a^)	NM_005916.5	Autosomal recessive	Missense/Nonsense	Compound heterozygous	Pathogenic/ pathogenic	MCM7-associated disease	Not OMIM listed
33	*H4C3*^a^	c.274 A > C, p.(Lys92Gln)	NM_003542.3	Autosomal dominant, de novo	Missense	Heterozygous	Pathogenic	Tessadori-Bicknell-van Haaften neurodevelop-mental syndrome 4	#619951
34	*ERF*	c.566_567del, p.(Cys189^a^)	NM_006494.2	Autosomal dominant, inherited	Frame-shift	Heterozygous	Pathogenic	Craniosynostosis 4	#600775
35	*BDNF*^a^	c.382 C > T, p.(Arg128Cys)	NM_001709.4	Autosomal dominant, de novo	Missense	Heterozygous	Likely pathogenic	BDNF-associated disorder	Not OMIM listed
	*Others*								
36	*DUOX2* *DUOX1*	c.1300 C > T, p.(Arg434^a^) c.1823-1 G > C, p.(?)	NM_014080.4 NM_017434.3	Autosomal recessive Autosomal recessive (modifier)	Nonsense	Homozygous Homozygous	Pathogenic Likely pathogenic	Thyroid dyshormonogenesis 6	#607200
37	*NF1* *PHEX*	c.226 G > T, p.(Glu76^a^) c.1303-2 A > G, p.(?)	NM_000267.3 NM_000444.6	Autosomal dominant inherited; X-linked, de novo	Nonsense Splice variant	Heterozygous Hemizygous	Pathogenic Pathogenic	Neurofibromatosis Type 1, Hypophosphatemic rickets, X-linked dominant	#162200 #307800

^a^Variants, which could be assigned to be disease-associated were reevaluated in multidisciplinary conferences in regard of phenotype, laboratory findings, and family history.

^b^At time of data interpretation unknown.

**Fig. 1:**
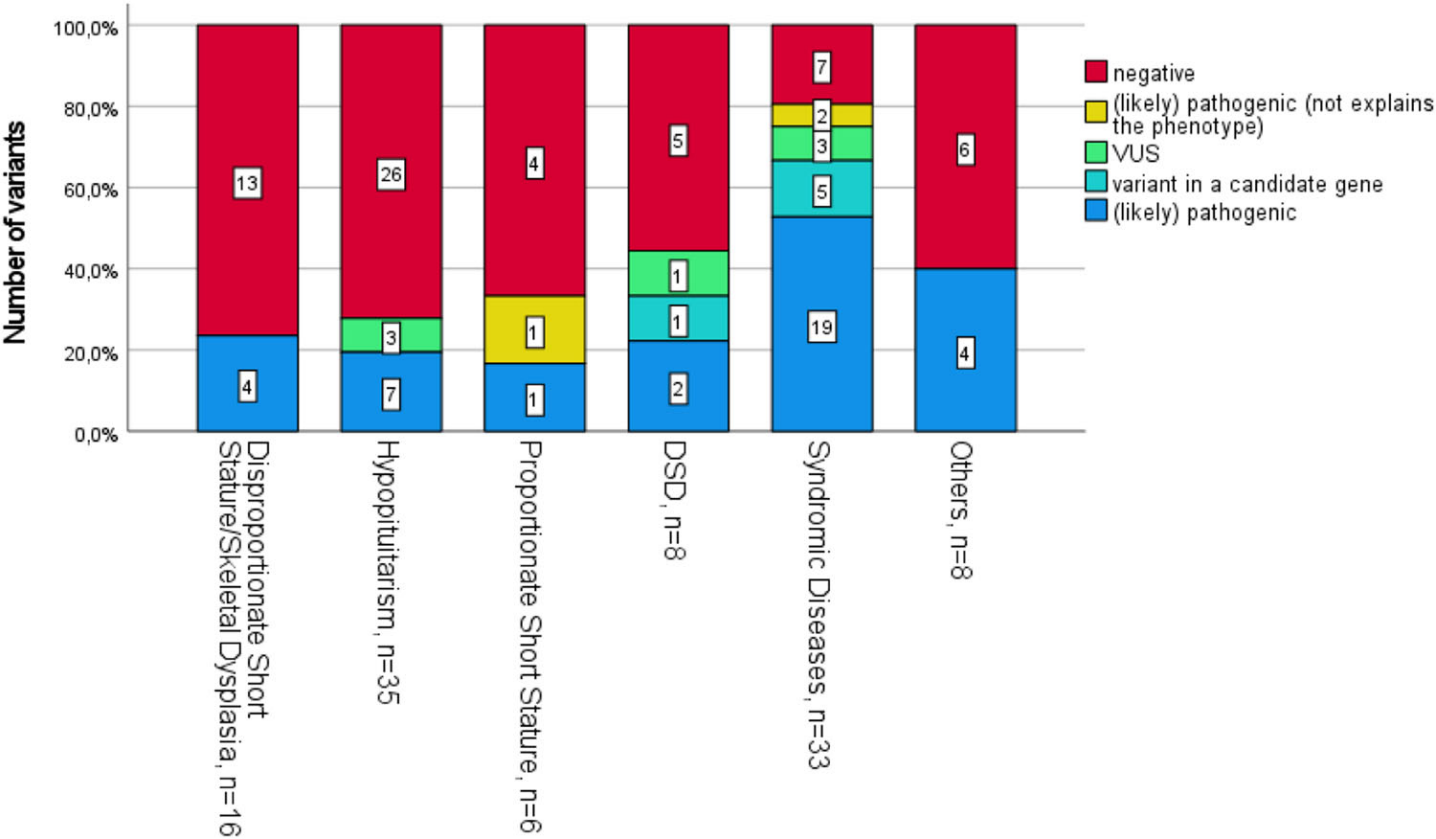
Variant classification of 106 patients with endocrine disorders. The bars show the distribution of variants according to the diagnoses. VUS variant of unknown significance, *DSD* disorder of sexual development*;* *n* = patients

The diagnostic yield significantly depended on the phenotype (*P* < 0.001, Fisher´s exact test). Figure 1 shows the detailed classification of the genetic variants at the time of testing in all subcategories. Variants of uncertain significance were intensively discussed in case conferences and classified accordingly, with 2 variants classified as likely benign (HESX1, Table 3) and 1 as benign (HSD3B2, Table 2). Remarkably, the diagnostic yield in the group of syndromic disorders (66.6%, 22/33) was significantly higher than in all other subcategories summarized (20.5% overall (15/73), Pearson chi-square 21.27, *P* < 0.001, cc=0.41). The diagnostic yield varied between the other subgroups: PSS: 16.6% (1/6); DSS: 18.8% (3/16); H: 17.1% (6/35); DSD: 37.5% (3/8); others: 25% (2/8) (Fig. 1). A total of 69/106 individuals (65.1%) remained unsolved after exome sequencing and multidisciplinary case conferences. The unsolved group included individuals with negative results (62/106, 58.5%), individuals with variants of uncertain significance (4/106, 3.8%) and variants not explaining the phenotype/secondary findings (2/106; 1.9%) (Table 3 and Supplementary Table 2).

**Table 3:** List of patients with variants not related to the phenotype* (*N* = 5) and secondary findings^ɫ^ (*N* = 2)

Patient	Gene/locus	Variant	Transcript	Inheritance	Variant type	Zygosity	Variant classification	Diagnosis	OMIM phenotype
	*Hypopituitarism*								
38 and 39	HESX1^*^	c.35 G > A, p.(Gly12Glu)	NM_003865.3	Autosomal dominant, inherited	Missense	Heterozygous	likely benign	Autosomal dominant and autosomal recessive inherited combined hypophyseal deficiency type 5	#182230
40	KMT2D^*^	c.15257 G > A, p.(Arg5086Gln)	NM_003482.3	Autosomal dominant, de novo	Missense	Heterozygous	VUS	Kabuki syndrome 1	#147920
	*Syndromic Diseases*								
41	FBN1^*^	c.2170 A > G, p.(Ile724Val)	NM_000138.4	Autosomal dominant, inherited	Missense	Heterozygous	VUS	Marfan syndrome	#154700
42	VPS13B^*^	c.11883_11885A [5], p.(Thr3963Argfs*51); c.1087 G > A, p.(Glu363Lys)	NM_017890.4	Autosomal recessive	Frameshift/Missense	Compound heterozygous	Pathogenic/ VUS	Cohen syndrome	#216550
	*Proportionate Short Stature*								
43	*Xq27.1*^ɫ^	chrX:137715011-138774283	-	X-linked, inherited	Deletion	Hemizygous	Pathogenic	Hemophilia B	#306900
	*Syndromic Diseases*								
44	*RNASEH2B*^ɫ^	c.529 G > A, p.(Ala177Thr)	NM_001142279.2	Autosomal recessive	Missense	Homozygous	Likely pathogenic	Aicardi-Goutieres syndrome 2	#610181

^*^indicates patients with variants not related to the phenotype

^ɫ^indicates secondary findings

### Inheritance

**Fig. 2:**
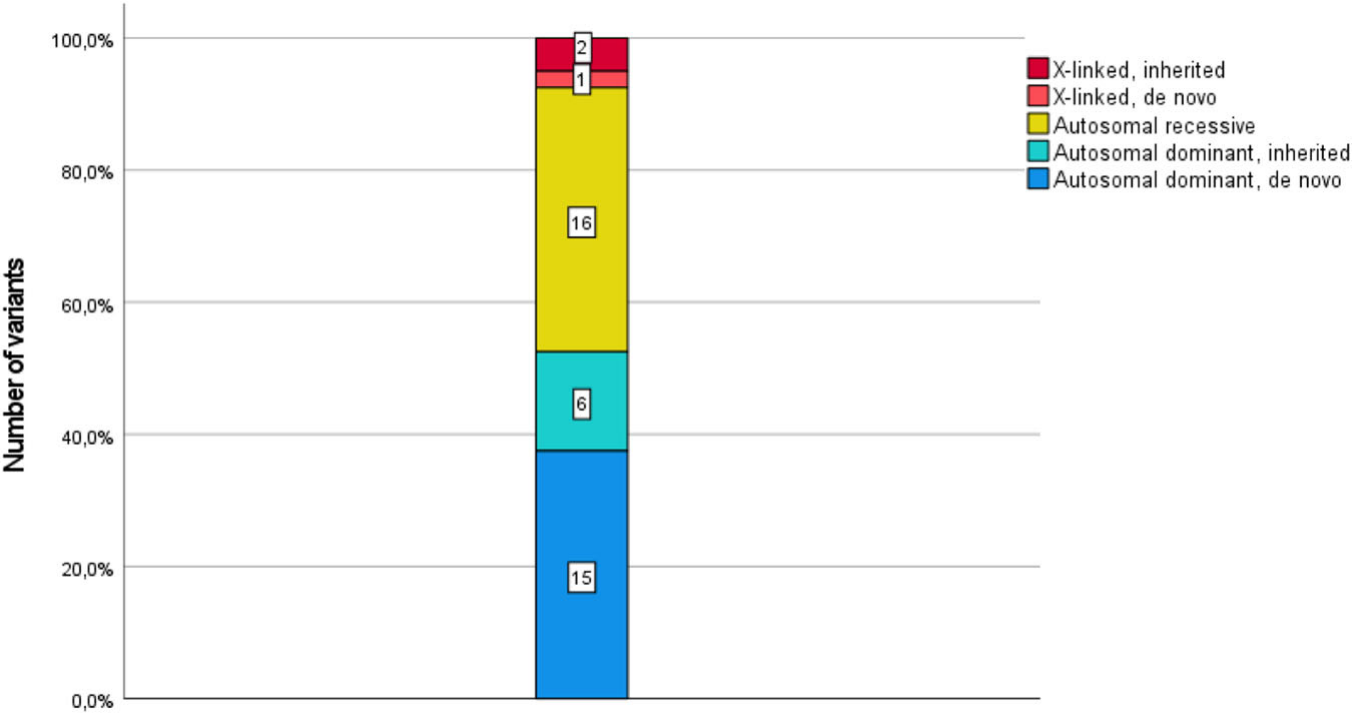
Distribution of all disease-causing variants based on the mode of inheritance, with de novo variants representing the most common mode of inheritance

Table 2 shows all 40 confirmed diagnoses in 37 patients, including three individuals with dual diagnoses. The majority of diagnoses (*n* = 16/40, 40%) were based on de novo variants in genes associated with either autosomal dominant disorders (*n* = 15/16, 93.7%) or with X-linked disorders (*n* = 1/16, 6.3%) (Table 2, Fig. 2). A total of 16/40 diagnoses (40%) were inherited in an autosomal recessive way with homozygous variants in *n* = 12/40 (30%) and compound heterozygous variants in *n* = 4/40 (10%) of patients. A total of 7/10 patients had a consanguineous background. In six autosomal dominant diagnoses, pathogenic variants were inherited from affected (*n* = 1) or unaffected (*n* = 5) parents, and three individuals received X-linked diagnoses (two patients with maternally inherited variants, one patient with a de novo variant) (Fig. 2).

### Novel gene-disease associations

In five individuals, variants were prioritized in genes that were not associated with monogenic disorders at the time of data interpretation. In the majority of these individuals (*n* = 4), de novo variants were found. Only one patient had compound heterozygous variants in the candidate gene *MCM7*. All variants were absent in gnomAD [11, 12]. All potential novel gene-disease associations were discussed by multidisciplinary teams and submitted to GeneMatcher [13, 14]. Three individuals were subsequently published within large collaborations linked by GeneMatcher [15–17]. A total of five novel gene-disease associations have been identified. Respective variants were found in *PPP1R12A* [15]*, SMARCA5* [16]*, H4C3* [18]*, MCM7*, and *BDNF*. Separate manuscripts are in preparation for these genes. As previously indicated, these five patients were considered solved and included in the overall yield. For patient characteristics, see Table 1 in the Supplementary Appendix.

### Secondary findings

In patient 43, a microdeletion of Xq27.1 affecting the *F9* gene was reported as a secondary finding, and the diagnosis of hemophilia was clinically confirmed (Table 3 and Supplementary Table 2). In patient 44, a homozygous missense variant (class 5), previously reported multiple times to be pathogenic, was reported as a secondary finding, as JAK inhibitors have been shown to be effective in the treatment of Aicardi-Goutieres syndrome [19]. The patient was subsequently clinically evaluated but showed no clinical signs of Aicardi-Goutieres syndrome, and neopterin in CSF was normal, as was the interferon signature. As the variant was identified in a healthy additional adult individual in a homozygous state in the in-house exome database, the multidisciplinary case conference concluded a reduced penetrance of *RNASEH2B*-associated Aicardi-Goutieres syndrome.

Sibling patients 38 and 39 with hypopituitarism harbored a likely benign variant in the *HESX1* gene, which was inherited from the unaffected mother. In patients 40, 41, 42 likely pathogenic variants or VUS were identified and reported back to the multidisciplinary teams. After intensive rephenotyping and discussion in case conferences, they were classified as “not disease-causing” (Table 3 and Supplementary Table 2).

### Dual diagnoses

Interestingly, we identified three patients with more than one genetic diagnosis. Patient 29 had a disease-causing variant in *WRN*, which explains the multiple features of accelerated aging in addition to microcephaly and mental retardation. A homozygous variant of uncertain significance was identified in *ASPM*, but the multidisciplinary board considered the diagnosis of primary autosomal recessive microcephaly 5 to be likely. Patient 36 had congenital primary hypothyroidism. In addition to a disease-causing variant in *DUOX2*, we also identified a homozygous splice variant in *DUOX1*. Given the unusual severe presentation, the multidisciplinary board classified the variant as a likely disease modifier. Patient 37 suffered from neurofibromatosis type 1 and *PHEX*-related hypophosphatemia. Patient characteristics are shown in Supplementary Table 2.

## Discussion

As more genetic alterations are discovered, particularly in ultrarare diseases, and next-generation sequencing becomes more widely available, we wanted to evaluate whether ES is a useful diagnostic tool in children and adolescents with endocrine disorders.

In an unselected cross-sectional cohort of 106 patients from a single endocrine center, ES identified likely pathogenic/pathogenic variants in 34.9% of previously undiagnosed patients with endocrine disorders. Of the confirmed diagnoses, 75% were ultrarare diseases. Here, we extend the list of disease-associated genes in 5 cases. An accurate phenotypic description including comprehensive endocrine diagnostics as well as the evaluation of variants in multidisciplinary case conferences involving geneticists are necessary for personalized diagnostic care and initiation of specific treatment, surveillance, and family counseling.

The achieved overall diagnostic yield of 34.9% was comparable to other published studies using ES in different phenotypes, [20–28] with a trend toward higher yields in complex phenotypes. The syndromic group showed a significantly higher diagnostic yield than the other groups (66.6% vs 20.5%).

A genetic diagnosis was established in 37 patients, including three patients with dual diagnosis, and causative variants were found in 40 genes. Most patients (*n* = 22) with a suspected underlying endocrine disorder were immediately substituted with appropriate hormones after pathological endocrine testing. After confirmation of the genetic diagnosis, these families were offered genetic counseling and were generally advised to continue hormone treatment. In one girl, hypophosphatemic rickets was confirmed as an unexpected secondary diagnosis, and treatment with burosumab was initiated. In three other patients, growth hormone treatment was started after the genetic diagnosis of Noonan syndrome and risk assessment together with the parents.

In certain syndromes, such as Noonan or Werner syndromes, individualized lifelong surveillance is recommended. In this cohort, extended surveillance was indicated in at least 7 patients (6.5%) after genetic diagnosis.

The majority of diagnoses were based on de novo variants or autosomal recessive inheritance (both 40%). The percentage of autosomal recessive disorders in our cohort was surprisingly high compared to previous studies in patients with neurodevelopmental disorders, which showed a low contribution (4–16%) of autosomal recessive disorders [26, 29]. In patients with endocrine disorders, the mode of inheritance has not been systematically investigated [22, 26].

The significant proportion of de novo variants highlights the utility of trio sequencing as a first-line strategy, especially in sporadic cases. Even in highly recognizable syndromes or defined endocrinopathies due to specific hormonal constellations, we discovered unexpected diagnoses. For example, patients with pathogenic variants in *NFKB2* or *IGSF1* or the dual diagnosis of patient 37 (neurofibromatosis and hypophosphatemic rickets) would have been missed by targeted panel sequencing. An important finding of this study is the high prevalence of ultrarare diseases (75.0%). In addition, we extended the list of disease-associated genes associated with endocrinopathies to facilitate variant classification in other patients. Three results have been published [15–17], and the others are in preparation for manuscript, highlighting once again the potential of international data sharing and collaboration [13, 14]. We strongly recommend interdisciplinary case conferences involving pediatric endocrinologists and geneticists to discuss patient phenotypes and known constellations together with genetic findings to classify new variants. Although controversial, some authors suggest the diagnostic yield as a parameter of effectiveness [27]. We believe that an individualized decision in a multidisciplinary case conference is preferable to avoid repetitive single gene or panel testing, as the cost of NGS techniques has decreased and this approach avoids the diagnostic odyssey of patients with complex rare diseases, thus anticipating personalized patient care and the most appropriate treatment. One limitation is that these results are derived from a single center. Therefore, the data should be confirmed in large international studies to evaluate the overall diagnostic yield in children and adolescents with endocrine disorders.

## Conclusions

The present study shows that ES is an effective tool for genetic diagnostics in pediatric patients with complex endocrine disorders. An accurate phenotypic description, including comprehensive endocrine diagnostics by pediatric endocrinologists together with variant evaluation by geneticists in multidisciplinary case conferences, is necessary for specific results and personalized clinical management. Furthermore, we were able to expand the list of disease-associated genes in 5 cases. For the first time, we estimated the diagnostic yield in different groups of endocrinopathies. It was highest in complex patients. Finally, the broad spectrum of genetic endocrinopathies, including ultrarare diseases in 75% of patients, was demonstrated, leading to the initiation of specific treatment, surveillance, and family counseling.

### Supplementary Information

Choukair suppl table 1

Choukair suppl table 2

STROBE_Choukair

## Data Availability

Additional data are available upon request from the corresponding author if in line with the consent.

## References

[1] Shlomo M., Auchus R.J., Goldfine A.B., Koenig R.J., Rosen C.J. Williams textbook of endocrinology. Philadelphia: Elsevier; 2020.

[2] Bundesausschuss IbG: TRANSLATE-NAMSE – Verbesserung der Versorgung von Menschen mit seltenen Erkrankungen durch Umsetzung von im nationalen Aktionsplan (NAMSE) konsentierten Maßnahmen. https://innovationsfonds.g-ba.de/projekte/neue-versorgungsformen/translate-namse-verbesserung-der-versorgung-von-menschen-mit-seltenen-erkrankungen-durch-umsetzung-von-im-nationalen-aktionsplan-namse-konsentierten-massnahmen.78 (2017). Accessed 20.01.2023.

[3] Kohler S, Gargano M, Matentzoglu N, Carmody LC, Lewis-Smith D, Vasilevsky NA (2021). The human phenotype ontology in 2021. Nucleic Acids Res.

[4] Riggs ER, Andersen EF, Cherry AM, Kantarci S, Kearney H, Patel A (2020). Technical standards for the interpretation and reporting of constitutional copy-number variants: a joint consensus recommendation of the American College of Medical Genetics and Genomics (ACMG) and the Clinical Genome Resource (ClinGen). Genet Med.

[5] Richards S, Aziz N, Bale S, Bick D, Das S, Gastier-Foster J (2015). Standards and guidelines for the interpretation of sequence variants: a joint consensus recommendation of the American College of Medical Genetics and Genomics and the Association for Molecular Pathology. Genet Med.

[6] Abou Tayoun AN, Pesaran T, DiStefano MT, Oza A, Rehm HL, Biesecker LG (2018). Recommendations for interpreting the loss of function PVS1 ACMG/AMP variant criterion. Hum. Mutat..

[7] Falb R.J., Muller A.J., Klein W., Grimmel M., Grasshoff U., Spranger S., et al. Bi-allelic loss-of-function variants in KIF21A cause severe fetal akinesia with arthrogryposis multiplex. J Med Genet. 2021. 10.1136/jmedgenet-2021-108064. 10.1136/jmedgenet-2021-108064 PMC9811090 34740919

[8] Granzow M, Paramasivam N, Hinderhofer K, Fischer C, Chotewutmontri S, Kaufmann L (2015). Loss of function of PGAP1 as a cause of severe encephalopathy identified by Whole Exome Sequencing: Lessons of the bioinformatics pipeline. Mol. Cell Probes.

[9] Freeman P, Hart R, Gretton L, Brookes A, Dalgleish R (2018). VariantValidator: Accurate validation, mapping and formatting of sequence variation descriptions. Hum. Mutat..

[10] Regulation (EU) No 536/2014 of the European Parliament and of the Council of 16 April 2014 on clinical trials on medicinal products for human use, and repealing Directive 2001/20/EC Text with EEA relevance. 2014. p. 1-76.

[11] Karczewski KJ, Francioli LC, Tiao G, Cummings BB, Alfoldi J, Wang Q (2020). The mutational constraint spectrum quantified from variation in 141,456 humans. Nature.

[12] MacDonald J., Ziman R., Yuen R., Feuk L., Scherer S. The database of genomic variants: a curated collection of structural variation in the human genome. Nucleic Acids Res. 2013. 10.1093/nar/gkt958 PMC3965079 24174537

[13] Sobreira N, Schiettecatte F, Boehm C, Valle D, Hamosh A (2015). New tools for Mendelian disease gene identification: PhenoDB variant analysis module; and GeneMatcher, a web-based tool for linking investigators with an interest in the same gene. Hum. Mutat..

[14] Sobreira N, Schiettecatte F, Valle D, Hamosh A (2015). GeneMatcher: a matching tool for connecting investigators with an interest in the same gene. Hum. Mutat..

[15] Hughes JJ, Alkhunaizi E, Kruszka P, Pyle LC, Grange DK, Berger SI (2020). Loss-of-Function Variants in PPP1R12A: From Isolated Sex Reversal to Holoprosencephaly Spectrum and Urogenital Malformations. Am. J. Hum. Genet.

[16] Li D., Wang Q., Gong N.N., Kurolap A., Feldman H.B., Boy N., et al. Pathogenic variants in SMARCA5, a chromatin remodeler, cause a range of syndromic neurodevelopmental features. Sci Adv. 2021;7(20). 10.1126/sciadv.abf2066. 10.1126/sciadv.abf2066 PMC8115915 33980485

[17] Tessadori F, Duran K, Knapp K, Fellner M, Smithson S, Deciphering Developmental Disorders S (2022). Recurrent de novo missense variants across multiple histone H4 genes underlie a neurodevelopmental syndrome. Am. J. Hum. Genet.

[18] Tessadori F, Duran K, Knapp K, Fellner M, Smithson S, Beleza Meireles A (2022). Recurrent de novo missense variants across multiple histone H4 genes underlie a neurodevelopmental syndrome. Am. J. Hum. Genet..

[19] Vanderver A, Adang L, Gavazzi F, McDonald K, Helman G, Frank DB (2020). Janus Kinase Inhibition in the Aicardi-Goutieres Syndrome. N. Engl. J. Med.

[20] Abreu AP, Dauber A, Macedo DB, Noel SD, Brito VN, Gill JC (2013). Central precocious puberty caused by mutations in the imprinted gene MKRN3. N. Engl. J. Med.

[21] Ji J., Shen L., Bootwalla M., Quindipan C., Tatarinova T., Maglinte D.T., et al. A semiautomated whole-exome sequencing workflow leads to increased diagnostic yield and identification of novel candidate variants. Cold Spring Harb Mol Case Stud. 2019;5(2). 10.1101/mcs.a003756. 10.1101/mcs.a003756 PMC6549575 30755392

[22] Retterer K, Juusola J, Cho MT, Vitazka P, Millan F, Gibellini F (2016). Clinical application of whole-exome sequencing across clinical indications. Genet Med.

[23] Srivastava S, Love-Nichols JA, Dies KA, Ledbetter DH, Martin CL, Chung WK (2019). Meta-analysis and multidisciplinary consensus statement: exome sequencing is a first-tier clinical diagnostic test for individuals with neurodevelopmental disorders. Genet Med.

[24] Tan TY, Dillon OJ, Stark Z, Schofield D, Alam K, Shrestha R (2017). Diagnostic Impact and Cost-effectiveness of Whole-Exome Sequencing for Ambulant Children With Suspected Monogenic Conditions. JAMA Pediatr..

[25] Tsang MHY, Chiu ATG, Kwong BMH, Liang R, Yu MHC, Yeung KS (2020). Diagnostic value of whole-exome sequencing in Chinese pediatric-onset neuromuscular patients. Mol. Genet Genom. Med.

[26] Brunet T, Jech R, Brugger M, Kovacs R, Alhaddad B, Leszinski G (2021). De novo variants in neurodevelopmental disorders-experiences from a tertiary care center. Clin. Genet.

[27] Smith HS, Swint JM, Lalani SR, Yamal JM, de Oliveira Otto MC, Castellanos S (2019). Clinical application of genome and exome sequencing as a diagnostic tool for pediatric patients: a scoping review of the literature. Genet Med.

[28] Newey PJ (2019). Clinical genetic testing in endocrinology: Current concepts and contemporary challenges. Clin. Endocrinol. (Oxf.).

[29] Martin HC, Jones WD, McIntyre R, Sanchez-Andrade G, Sanderson M, Stephenson JD (2018). Quantifying the contribution of recessive coding variation to developmental disorders. Science.

