# Supplementary material for: Exome sequencing (ES) of a pediatric cohort with chronic endocrine diseases: a single-center study (within the framework of the TRANSLATE-NAMSE project)
Source: Endocrine. 2023 Nov 8;85(1):444–53. doi: 10.1007/s12020-023-03581-7 (PMC11246252; doi:10.1007/s12020-023-03581-7)
Supplement: Supplementary file 1 — Choukair suppl table 1 [file 12020_2023_3581_MOESM1_ESM.docx]

Supplementary Table 1: List of patients in whom the variants were found to be associated with the disease (n = 37)

| Patient | Gene/locus | Variant | Hormonal deficiencies | Phenotype |
| --- | --- | --- | --- | --- |
|  | Disproportionate Short Stature/  Skeletal Dysplasia |  |  |  |
| 1 | *ANKRD11* | c.7534C>T, p.(Arg2512Trp) | - | 5-year-old girl with disproportionate short stature, combined developmental delay, trigonocephaly, tethered cord |
| 2 | *PAPSS2* | c.809G>A, p.(Gly270Asp) | DHEA-S | 18-year-old boy with disproportionate short stature, small for gestational age, hexadactyly, coxa retrotorta left, low arch and flatfeet |
| 3 | *MBTPS1* | c.1995C>G, p.(Tyr665*),  c.955G>T, p.(Val319Phe) | - | 13-year-old boy with disproportionate short stature, proximal accentuated muscle weakness, lordosis |
|  | Hypopituitarism |  |  |  |
| 4 | *GNRHR* | c.317A>G, p.(Gln106Arg); c.350T>G, p.(Leu117Arg) | LH, FSH | 21-year-old man with isolated hypogonadotropic hypogonadism, no anosmia |
| 5 | *FGFR1* | c.1704+1G>A, p.(?) | LH, FSH | 20-year-old woman with isolated hypogonadotropic hypogonadism, no anosmia |
| 6 | *NFKB2* | c.2600C>T, p.(Ala867Val) | ACTH | 18-year-old boy with isolated secondary adrenal insufficiency, no history of recurrent infections |
| 7 | *IGSF1* | c.2422dup, p.(His808Profs*14) | TSH, PRL | 5-year-old boy with secondary hypothyroidism, adiposity, macrocephaly, developmental delay |
| 8 und 9 | *FGFR1*  Proportionate Short Stature | c.287C>G,  p.(Ser96Cys) | LH, FSH | 20- and 22-year-old women with isolated hypogonadotropic hypogonadism, no anosmia |
| 10 | *GHR* | c.344A>C, p.(Asn115Thr) | IGF-I | 13-year-old boy with severe proportionate short stature, marked IGF-I deficiency, supraphysiological GH increase on arginine-and insulin-tolerance-test |
|  | Differences of Sex Development |  |  |  |
| 11 | *HSD3B2* | c.500C>T, p.(Ala167Val), c.946C>T, p.(Arg316Cys) | - | 3-year-old boy with proximal hypospadias and micropenis, undescended testes, macrocephaly, muscular hypotonia, elevated 17-OH-pregnenolone/17-OH-progesterone ratio, sufficient cortisol secretion in co-syntropin-stimulation- test, karyotype, XY |
| 12 | *PPP1R12A** | c.2698C>T, p.(Arg900*) | - | 4-year-old phenotypic female with a 46, XY normal male karyotype, clitoral hypertrophy (0.5 cm in diameter), urogenital sinus (UGS), vaginal opening and posterior fusion of the labia majora. No uterus identified on pelvic ultrasound. Elevated FSH, estradiol undetectable, testosterone detectable, but too low at the age of so-called “mini-puberty”, AMH and Inhibin B detectable and suggestive of testicular gonads |
| 13 | *AR* | c.2495G>A, p.(Arg832Gln) | - | 4-year-old phenotypic female with a 46, XY normal male karyotype with separated urethra and vaginal opening, no uterus identified on pelvic ultrasound, bilateral hernia inguinales with gonads. Elevated AMH detectable, indicating functioning Sertoli cells. |
|  | Syndromic Diseases |  |  |  |
| 14 | *ABL1* | c.1066G>A, p.(Ala356Thr) | - | 20-year-old woman with growth failure, right kidney dysplasia, neurogenic bladder voiding dysfunction, Arnold-Chiari malformation, multiple VSDs, persistent D. art. Botalli, pulmonary hypertension, microcephaly |
| 15 | *ALMS1* | c.4150dup, p.(Thr1384Anfs*15) | - | 21-year-old woman with short stature, retinal dystrophy, obesity, chronic renal failure, arterial hypertension, hypertriglyceridemia, empty sella, deafness |
|  |  |  |  |  |
| 16 | *RAD21* | c.3G>A, p.(?) | - | 16-year-old girl with short stature, microcephaly, VSD, short, upturned nose, malformed ears |
| 17 | *FKBP14* | c.636G>C, p.(Ter212Tyrext*52) | - | 6-year-old girl with growth failure, muscle hypotonia, joint hypermobility, myopia, microcephaly |
| 18 | *PSMD12* | c.148_149del, p.(Leu50Glyfs*26) | - | 4-year-old boy with growth failure, hypotrophic newborn at 29+2 weeks of gestation, VSD, right-sided aortic arch, proximal hypospadias and micropenis, bilateral renal dysplasia , anal atresia |
| 19 | *PIBF1* | c.1133A>C, p.(His378Pro), c.1801C>T, p.(Arg601*) | LH/FSH, TSH, ACTH, GH | 22-year-old man with chronic renal failure III, complex brain malformations (cerebellar dysplasia, underdevelopment of the cerebellar vermis, pituitary agenesis, polymicrogyria of the left parietal side), panhypopituitarism, psychomotor retardation, myofibroblastic tumor of the omentum majus, liver fibrosis with portal hypertension and esophageal varices |
| 20 | *SMARCA5** | c.1301_1306del, p.(Ile434_Leu435del) | - | 13-year-old boy with short stature, born small for gestational age, failure to thrive with microcephaly and mild developmental delay, hypogammaglobulinemia |
| 21 | *POLD1* | c.1812_1814del, p.(Ser605del) | - | 28-year-old man with hypogonadism, lipid dystrophy, bone mineralization disorder, muscular dystrophy, hyperkeratosis at both feet, polyneuropathy, deafness |
| 22 | *BRAF* | c.1741A>G, p.(Asn581Asp) | - | 6-year-old boy with growth failure, hypertrophic newborn, muscular hypotonia, low arches and flat feet, facial features: large, low set ears with wide helix, missing eyebrows and eyelashes, steel blue eyes, hypertelorism, speech development delay |
| 23 | *PTPN11* | c.922A>G, p.(Asn308Asp) | GH | 14-year-old boy with short stature, GH-deficiency, low set ears, thrombocytopenia |
| 24 | *PTPN11* | c.794G>A, p.(Arg265Gln) | - | 5-year-old boy with short stature, hypotrophic newborn in the 30^th^ week of gestation, global developmental delay, microcephaly, stenosis of the right pulmonary artery, strabismus, myopia and astigmatism |
| 25 | *DNAJC21* | c.647_666del, p.(Arg216Thrfs*63) | GH | 18-year-old boy with short stature, obesity, GH-deficiency, scoliosis, developmental delay, cryptorchism, clubfeet |
| 26 | *PUF60* | c.1172_1173insATA, p.(Val392*) | - | 10-year-old girl with short stature, small for gestational age, global developmental delay, astigmatism, brachymesophalangia D. V on both hands |
| 27 and 28 | *ADAMTS10* | c.709C>T, p.(Arg237*) | GH  - | 27: 9-year-old boy with combined developmental delay, short fingers, small plumb hands and toes, low set ears, decent epicanthus, microcephaly, short stature with growth hormone deficiency, hypoplasia of the adenohypophysis, congenital combined mitral valve disease with significant mitral stenosis and moderate mitral regurgitation, spontaneous closure of a muscular ventricular septal defect (VSD), partially compensated heart failure on anti-congestive therapy  28: 5-year-old girl with short stature, congenital combined mitral valve disease with insufficiency and stenosis, grade III vesicourethral reflux disease |
| 29 | *WRN*  *ASPM* | c.3913C>T, p.(Arg1305*)  c.2474G>A, p.(Arg825Gln) | -  - | 24-year-old man with congenital hypothyroidism with thyroid hypoplasia, short stature, progeroid symptoms such as early-onset of arterial hypertension, diabetes mellitus type II, early adrenarche, hair loss, microcephaly, low intelligence, developmental delay |
| 30 | *KMT2A* | c.1844del, p.(Pro615Argfs*8) | - | 9-year-old girl with obesity, premature adrenarche, mild mental retardation, facial features: hypertelorism, long eyelashes, short philtrum; large and broad hands, mild muscular hypotonia |
| 31 | *KMT2D* | c.10624C>G, p.(Leu3542Val) | GH, LH/FSH, ACTH, TSH, PRL | 19-year-old woman with bilateral choanal atresia, left auricular dysplasia, left microtia II°, congenital stenosis of the left auditory canal, amastia, agenesis of the gallbladder, renal hypoplasia with congenital renal insufficiency, an anterior anus position and panhypopituitarism with complete agenesis of the adenohypophysis, diabetes mellitus (insulin-dependent) |
| 32 | *MCM7** | c.776G>C, p.(Gly259Ala); c.133C>T, p.(Gln45*) | - | 6-year-old girl with pseudopubertas praecox due to a left adrenal cortical adenoma, short stature, hypotrophic newborn, psychomotor retardation, microcephaly, curly hair, lipodystrophy, facial anomalies such as progeroid appearance, enophthalmos, trichiasis, hyperopia, astigmatism, anisometropia, retrognathia |
| 33 | *H3C4** | c.274A>C, p.(Lys92Gln) | - | 4-year-old boy with intrauterine growth retardation, short stature, microcephaly, muscular hypotonia, joint hypermobility, combined developmental delay with almost no expressive speech and moderate mental retardation due to frontotemporal brain atrophy, VSD, hypospadias, undescended testes and facial features: ptosis, hypertelorism, inverted epicanthus, oblique eyelid axes, very light and curved eyebrows, depressed nasal root, broad nasal tip with anteverted nares, low set ears, wide mouth and widely spaced teeth |
| 34 | *ERF* | c.566_567del, p.(Cys189*) | - | 5-year-old boy with short stature, combined developmental delay, Arnold-Chiari I malformation with deep tonsils, facial features: hypertelorism, low hairline, increased facial vein markings with prominent cranial sutures |
| 35 | *BDNF** | c.382C>T, p.(Arg128Cys) | - | 13-year-old girl with growth failure, obesity after six months of life, lack of satiety, learning disability, intelligence slightly below average range, developmental disorder of motor function, intermittent generalized idiopathic epilepsy (absence epilepsy), behavioral problems with strong oppositional behavior |
|  | Others |  |  |  |
| 36 | *DUOX2*  *DUOX1* | c.1300C>T, p.(Arg434*)  c.1823-1G>C, p.(?) | TSH | 4-year-old girl with congenital, primary hypothyroidism |
| 37 | *NF1*  *PHEX* | c.226G>T, p.(Glu76*)  c.1303-22A>G, p.(?) | -  - | 17-year-old girl with short stature, neurofibromatosis, unilateral optic nerve glioma, unilateral renal cysts, tubulopathy with phosphate wasting, arterial hypertension, crus varum |
|  |  |  |  |  |

*Variants, which could be assigned to be disease associated were in multidisciplinary conferences reevaluated in regard of phenotype, laboratory findings, and family history
