# Supplementary material for: Exome sequencing (ES) of a pediatric cohort with chronic endocrine diseases: a single-center study (within the framework of the TRANSLATE-NAMSE project)
Source: Endocrine. 2023 Nov 8;85(1):444–53. doi: 10.1007/s12020-023-03581-7 (PMC11246252; doi:10.1007/s12020-023-03581-7)
Supplement: Supplementary file 2 — Choukair suppl table 2 [file 12020_2023_3581_MOESM2_ESM.docx]

Supplementary Table 2: List of patients with variants not related to the phenotype^*^ (n = 5) and secondary findingsᶧ (n=2)

| Patient | Gene/locus | Variant | Hormonal deficiencies | Phenotype |
| --- | --- | --- | --- | --- |
|  | Hypopituitarism |  |  |  |
| 38 and 39 | *HESX1***^*^** | c.35G>A, p.(Gly12Glu) | GH, ACTH, TSH, LH/FSH | 38: Congenital hypopituitarism with cranially ectopic neurohypophysis, subtotal absence of infundibulum, extremely small adenohypophysis in very slender sella, hypoglycaemia in newborn age  39: Congenital hypopituitarism with malposition of the sella, small cone of tissue possibly corresponding to adeno-pituitary tissue, no neurohypophysis and no infundibulum detectable |
| 40 | *KMT2D***^*^** | c.15257G>A, p.(Arg5086Gln) | ACTH | Postnatal secondary adrenal insufficiency with hypoglycaemia, reconstitution at 5 months of age, age-appropriate development |
|  |  |  |  |  |
|  | Syndromic  Diseases |  |  |  |
| 41 | *FBN1***^*^** | c.2170A>G, p.(Ile724Val) |  | Flat feet, brachydactyly type E2, ADHD, dystrophy, constitutional tall stature, trivial mitral regurgitation with minor mitral valve prolapse, facial features such as narrow face, deep set, slightly protruding ears |
| 42 | *VPS13B***^*^** | c. 11883_11885A[5], p.(Thr3963Argfs*51); c.1087G>A, p.(Glu363Lys) |  | Short stature, global developmental delay, ADHD, recurrent constipation with faecal incontinence, minimal left convex scoliosis |
|  | Proportionate Short Stature |  |  |  |
| 43 | *Xq27.1***ᶧ** | chrX:137715011-138774283 | - | Haemophilia B ("moderate"; residual F IX activity 1.1%), pulsatile stenosis above the tracheal bifurcation with subtotal obstruction of the left main bronchus, short stature, failure to thrive, slight hypertrichosis |
|  | Syndromic Diseases |  |  |  |
| 44 | *RNASEH2B***ᶧ** | c.529G>A, p.(Ala177Thr) | - | Primary congenital hypothyroidism, persistent foramen ovale, age-appropriate development, decent abnormalities with white substance deficit, myelination delay, and onset of signaling changes in the periventricular white substance, normal neopterin and interferon signature in CSF |
|  |  |  |  |  |

*ADHD: attention deficit hyperactive disorder; F: factor; CSF: cerebrospinal fluid*
